# Supplementary material for: A dCas9 proximity reporter for investigating DNA linear and rotational dynamics
Source: PLoS One. 2026 Oct 29;21(10):e0355748. doi: 10.1371/journal.pone.0355748 (PMC13719016; doi:10.1371/journal.pone.0355748)
Supplement: Supplementary file 1 [file pone.0355748.s001.pdf]

Supplementary Information to Accompany
A dCas9 Proximity Reporter for Investigating DNA Linear and Rotational Dynamics

Saba Parvez[1,†], Morgan C. Marsh[2,†], Shawn C. Owen[2,3,*], Randall T. Peterson[4,*],

1. Department of Cell & Developmental Biology, Northwestern University Feinberg School of Medicine
2. Department of Molecular Pharmaceutics, University of Utah
3. Department of Medicinal Chemistry; Department of Biomedical Engineering, University of Utah
4. Department of Pharmacology and Toxicology, University of Utah

† equal contributions

*corresponding authors: shawn.owen@hsc.utah.edu; randall.peterson@pharm.utah.edu

S1 Table. Primer Sequences For Bacterial Expression Plasmid Cloning

**Primer Designation**	**Primer Sequence (5'-3')**
dCas9-TEV-6xHis-fwd-1	gtttaactttaagaaggagatatacat ATG GATAAGAAATACTCAATAGGCTTAGCTATC
dCas9-TEV-6xHis-rev-1	TGCCCTGAGCCCTGGAAATACAAGTTTTCCGAGCCGTCACCTCCTAGCTGACTCAAATCA
dCas9-TEV-6xHis-fwd-2	caattcccctctagaaataattttgt ttaactttaagaaggagatatacat ATG GATAAG
dCas9-TEV-6xHis-rev-2	GGCTTTGTTAGCAGCCGGATCTCAGTGGTGGTGGTGGTGGTGCCCTGAGCCCTGGAAATA
dCas9-TEV-6xHis-fwd-3	tataggggaattgtgagcggataa caattcccctctagaaataattttgt
dCas9-TEV-6xHis-rev-3	TTATTGCTCAGCGGTGGCAGCAGCCAACTCAGCTTCCTTTCGGGCTTTGTTAGCAGCCGG
lucβ9-(GGGSG)3x-PCP-TEV-6xHis-fwd-1	CAGGT GGGGGAGGTAGCGGT GGAGGGGGTTCAGGG atgtccaaaaccatcgttctttc
lucβ9-(GGGSG)3x-PCP-TEV-6xHis-rev-1	TGCCCTGAGCCCTGGAAATACAAGTTTTCCGAGCCACGGCCCAGCGGCACAAG
lucβ9-(GGGSG)3x-PCP-TEV-6xHis-fwd-2	GTAGTATGCTGTTTCGTGTTACCATTAACAGC GGTGGTGGTTCAGGT GGGGGAGGTAGCG
lucβ9-(GGGSG)3x-PCP-TEV-6xHis-rev-2	GGCTTTGTTAGCAGCCGGATCTCAGTGGTGGTGGTGGTGGTGCCCTGAGCCCTGGAAATA

lucβ9-(GGGSG)3x-PCP-TEV-6xHis-fwd-3	tttgtttaactttaagaaggagatatacatatgGGTAGTATGCTGTTTCGTGTTACCATT
lucβ9-(GGGSG)3x-PCP-TEV-6xHis-rev-3	TTATTGCTCAGCGGTGGCAGCAGCCAACTCAGCTTCCTTTCGGGCTTTGTTAGCAGCCGG
lucβ10-(GGGSG)3x-MCP-TEV-6xHis-fwd-1	TTCAGGT GGGGGAGGTAGCGGT GGAGGGGGTTCAGGG atggcttctaactttactcagttcg
lucβ10-(GGGSG)3x-MCP-TEV-6xHis-rev-1	TGCCCTGAGCCCTGGAAATACAAGTTTTCCGAGCCGTAGATGCCGGAGTTTGCTG
lucβ10-(GGGSG)3x-MCP-TEV-6xHis-fwd-2	GTGAGCGGTTGGCGTCTGTTCAAAAAAATCAGC GGTGGTGGTTCAGGT GGGGGAGGTAGC
lucβ10-(GGGSG)3x-MCP-TEV-6xHis-rev-2	tttgtttaactttaagaaggagatatacatatgGGTAGTATGCTGTTTCGTGTTACCATT
lucβ10-(GGGSG)3x-MCP-TEV-6xHis-fwd-3	gaaataattttgtttaactttaagaaggagatatacatatgGTGAGCGGTTGGCGTCTGT
lucβ10-(GGGSG)3x-MCP-TEV-6xHis-rev-3	TTATTGCTCAGCGGTGGCAGCAGCCAACTCAGCTTCCTTTCGGGCTTTGTTAGCAGCCGG
SP6-gRNA-MS2-fwd	ATTTAGGTGACACTATA GATGGCATTGGTGGATTAAG Gtttaagagctatgctggaaaca
SP6-gRNA-2xMS2-rev	AAAAAAAGGGAACACGAGCG
SP6-gRNA-PP7-fwd	ATTTAGGTGACACTATA GGTCAGTGGTGGAGCCGGTC Gtttaagagctatgctggaaaca
SP6-gRNA-2xPP7-rev	AAAAAAAGGGAACTGCTGCG
SP6-gRNA-Sirius8x-rev	AAAAAAAGCACCGACTCGGT
SP6-gRNA-8xaptamer-rev	AAAAAAAGTGCCATGTTTCATCC
