# Supplementary material for: A dCas9 proximity reporter for investigating DNA linear and rotational dynamics
Source: PLoS One. 2026 Oct 29;21(10):e0355748. doi: 10.1371/journal.pone.0355748 (PMC13719016; doi:10.1371/journal.pone.0355748)
Supplement: Supplementary file 2 [file pone.0355748.s002.pdf]

S2 Table. Primer Sequences for Mammalian Expression Plasmid Cloning	
**Primer**	**Sequence**
pSICO-b10 fwd	Gctggtttagtgaaccgtca gatccgctagcccacc ATGGTGAGCGGTTGGCGT
MCP-2XFlag rev	AATCactaccCTTGTCGTCATCGTCTTTGTAGTCactacc GTAGATGCCGGAGTTTGCTG
pSICO ext1 fwd	Gggcggtaggcgtgtacggt gggaggtctatataaGcaga Gctggtttagtgaaccgtca
2xFlag-NLS ext 1 rev	cttttctttttgggactaccTTTATCGTCATCATCCTTGT AATCactaccCTTGTCGTCA
pSICO ext2 fwd	Ttccaaaatgtcgtaacaac tccgccccattgacgcaaat Gggcggtaggcgtgtacg
NLS-pSICO ext2 rev	tagatgcatgctcgacccgggTCAcacttttcttttctttttgggactaccTTTATCGTC
pSICO ext3 fwd	Ggagtttgttttggcaccaaaatcaacgggact Ttccaaaatgtcgtaacaactcc
pSICO ext3 rev	acgttaggggggggggagggagaggggcggaattggccgccctagatgcatgctcgaccc
pSICO-lucb9 fwd	Gctggtttagtgaaccgtca gatccgctagcccacc atgGGTAGTATGCTGTTTCGTGTTACCATTA
PCP-2xFlag rev	GTAATCactaccCTTGTCGTCATCGTCTTTGTAGTCactaccacggcccagcggcacaa
Linker-Δ11S fwd	gacgataagggtggcgggtccggcggtggatccggt ATGGTTTTTACCCTGGAAGATTTT
Δ11S-pcDNA3 rev	tttaaactcattactaaccggtagggatcgaaccctTTTAATCCGGTGTAATCAGACGTT
3xFlag ext fwd	acggagactacaaggatcatgatattgattacaaagacgat GACGATAAGGGTGGCGGGT
pcDNA3 ext rev	gtctccttccgtgtttcagttagcctcccccgTTTAAACTCATTACTAACCGGTAGGGAT
SV40-NLS-Flag ext fwd	aggtgggaggctccggaggaagcgactataaggaccacg ACGGAGACTACAAGGATCATG
pcDNA3 ext2 rev	ctttttattgccgtcatagcgcgggttccttccggtattGTCTCCTTCCGTGTTTCAGTT
bstXI-Alu-sgRNA1 fwd	cttggagaa CCACCTTGTTG G AGCACTTTGGGAGGCCGAGGC Gtttaagagctatgctggaaaca
sgRNA-XhoI rev	GATCCTAGTACTCGAGACCGAACTC
sgRNA-NotI rev	CGGTAATACGGTTATCCACG
bstXI-Alu-sgRNA2 fwd	cttggagaa CCACCTTGTTG GGGGTTTCACCATGTTGGCC Gtttaagagctatgctggaaaca
bstXI-cont-sgRNA1 fwd	cttggagaa CCACCTTGTTG GTATTACTGATATTGGTGGG Gtttaagagctatgctggaaaca
bstXI-cont-sgRNA2 fwd	cttggagaa CCACCTTGTTG GACCACTAATGAGATTCTTGT Gtttaagagctatgctggaaaca
bstXI-Alu-sgRNA3 fwd	cttggagaa CCACCTTGTTG CCCAGCACTTTGGGAGGCCG Gtttaagagctatgctggaaaca
bstXI-Alu-sgRNA4 fwd	cttggagaa CCACCTTGTTG GAATGGCGTGAACCCGGGAGG Gtttaagagctatgctggaaaca
bstXI-Alu-sgRNA5 fwd	cttggagaa CCACCTTGTTG GTCTCGCTCTGTCGCCCAGGC Gtttaagagctatgctggaaaca
