# Supplementary material for: A dCas9 proximity reporter for investigating DNA linear and rotational dynamics
Source: PLoS One. 2026 Oct 29;21(10):e0355748. doi: 10.1371/journal.pone.0355748 (PMC13719016; doi:10.1371/journal.pone.0355748)
Supplement: Supplementary file 3 [file pone.0355748.s003.pdf]

**S3 Table. Protein Sequences**	
**Protein**	**Sequence**
dCas9-<br>TEV-<br>6xHis	MDKKYSIGLAIGTNSVGWAVITDEYKVPSKKFKVLGNTDRHSIKKNLIGALLFDS<br>GETAEATRLKRTARRRYTRRKNRICYLQEIFSNEMAKVDDSFFHRLEESFLVEED<br>KKHERHPIFGNIVDEVAYHEKYPTIYHLRKKLVDSTDKADLRLIYLALAHMIKFR<br>GHFLIEGDLNPDNSDVDKLFIQLVQTYNQLFEENPINASGVDAKAILSARLSKSRR<br>LENLIAQLPGEKKNGLFGNLIALSLGLTPNFKSNFDLAEDAKLQLSKDTYDDDLD<br>NLLAQIGDQYADLFLAAKNLSDAILLSDILRVNTEITKAPLSASMIKRYDEHHQD<br>LTLLKALVRQQLPEKYKEIFFDQSKNGYAGYIDGGASQEEFYKFIKPILEKMDGT<br>EELLVKLNREDLLRKQRTFDNGSIPHQIHLGELHAILRRQEDFYPFLKDNREKIEK<br>ILTFRIPYYVGPLARGNSRFAWMTRKSEETITPWNFEEVVDKGASAQSFIERMTN<br>FDKNLPNEKVLPKHSLLYEYFTVYNELTKVKYVTEGMRKPAFLSGEQKKAIVDL<br>LFKTNRKVTVKQLKEDYFKKIECFDSVEISGVEDRFNASLGTYHDLLKIIKDKDF<br>LDNEENEDILEDIVLTLTLFEDREMIEERLKTYAHLFDDKVMKQLKRRRYTGWG<br>RLSRKLINGIRDKQSGKTILDFLKSDGFANRNFMQLIHDDSLTFKEDIQKAQVSG<br>QGDSLHEHIANLAGSPAIKKGILQTVKVVDELVKVMGRHKPENIVIEMARENQT<br>TQKGQKNSRERMKRIEEGIKELGSQILKEHPVENTQLQNEKLYLYYLQNGRDMY<br>VDQELDINRLSDYDVDAIVPQSFLKDDSIDNKVLTRSDKNRGKSDNVPSEEVVK<br>KMKNYWRQLLNAKLITQRKFDNLTKAERGGLSELDKAGFIKRQLVETRQITKHV<br>AQILDSRMNTKYDENDKLIREVKVITLKSKLVSDFRKDFQFYKVREINNYHHAH<br>DAYLNAVVGTALIKKYPKLESEFVYGDYKVYDVRKMIAKSEQEIGKATAKYFF<br>YSNIMNFFKTEITLANGEIRKRPLIETNGETGEIVWDKGRDFATVRKVLSMPQVNI<br>VKKTEVQTGGFSKESILPKRNSDKLIARKKDWDPKKYGGFDSPTVAYSVLVVAK<br>VEKGKSKKLKSVKELLGITIMERSSFEKNPIDFLEAKGYKEVKKDLIIKLPKYSLF<br>ELENGRKRMLASAGELQKGNELALPSKYVNFLYLASHYEKLKGSPEDNEQKQL<br>FVEQHKHYLDEIIEQISEFSKRVILADANLDKVLSAYNKHRDKPIREQAENIIHLFT<br>LTNLGAPAAFKYFDTTIDRKRYTSTKEVLDATLIHQSITGLYETRIDLSQLGGDGS<br>ENLYFQGSGHHHHHH-
lucβ9-<br>(GGGSG)<br>3x-MCP-<br>TEV-<br>6xHis	MGSMLFRVTINSGGGSGGGGSGGGGSGMASNFTQFVLVDNGGTGDVTVAPSNF<br>ANGIAEWISSNSRSQAYKVTCSVRQSSAQNRKYTIKVEVPKGAWRSYLNMELTI<br>PIFATNSDCELIVKAMQGLLKDGNPIPSAIAANSGIYGSENLYFQGSGHHHHHH
lucβ10-<br>(GGGSG)<br>3x-PCP-<br>TEV-<br>6xHis	MVSGWRLFKKISGGGSGGGGSGGGGSGMSKTIVLSVGEATRTLTEIQSTADRQIF<br>EEKVGPLVGRLRLTASLRQNGAKTAYRVNLKLDQADVVDSGLPKVRYTQVWS<br>HDVTIVANSTEASRKSLYDLTKSLVATSQVEDLVVNLVPLGRGSENLYFQGSGH<br>HHHHH
