# Supplementary material for: A dCas9 proximity reporter for investigating DNA linear and rotational dynamics
Source: PLoS One. 2026 Oct 29;21(10):e0355748. doi: 10.1371/journal.pone.0355748 (PMC13719016; doi:10.1371/journal.pone.0355748)
Supplement: Supplementary file 4 [file pone.0355748.s004.pdf]

S4 Table. DNA Sequences for the PAM-IN Orientation	
**Base Separation**	**DNA Sequence**
4	TCCGACACGTCCGATT GATGGCATTGGTGGATTAAGCGG atgg CCTGACCGGCTCCACCACTGACC TAAGCTGGAACAGACG
9	TCCGACACGTCCGATT GATGGCATTGGTGGATTAAGCGG atgg tgagc CCTGACCGGCT CCACCACTGACC TAAGCTGGAACAGACG
14	TCCGACACGTCCGATT GATGGCATTGGTGGATTAAGCGG atgg tgagc aaggg CCTGACCGGCTCCACCACTGACC TAAGCTGGAACAGACG
19	TCCGACACGTCCGATT GATGGCATTGGTGGATTAAGCGG atgg tgagc aaggg cgagg CCTGACCGGCTCCACCACTGACC TAAGCTGGAACAGACG
24	TCCGACACGTCCGATT GATGGCATTGGTGGATTAAGCGG atgg tgagc aaggg cgagg aggat CCTGACCGGCTCCACCACTGACC TAAGCTGGAACAGACG
29	TCCGACACGTCCGATT GATGGCATTGGTGGATTAAGCGG atgg tgagc aaggg cgagg aggat aacat CCTGACCGGCT CCACCACTGACC TAAGCTGGAACAGACG
30	TCCGACACGTCCGATT GATGGCATTGGTGGATTAAGCGG atgg tgagc aaggg cgagg aggat aacat g CCTGACCGGCT CCACCACTGACC TAAGCTGGAACAGACG
31	TCCGACACGTCCGATT GATGGCATTGGTGGATTAAGCGG atgg tgagc aaggg cgagg aggat aacat gg CCTGACCGGCT CCACCACTGACC TAAGCTGGAACAGACG
32	TCCGACACGTCCGATT GATGGCATTGGTGGATTAAGCGG atgg tgagc aaggg cgagg aggat aacat ggc CCTGACCGGCT CCACCACTGACC TAAGCTGGAACAGACG
33	TCCGACACGTCCGATT GATGGCATTGGTGGATTAAGCGG atgg tgagc aaggg cgagg aggat aacat ggcc CCTGACCGGCT CCACCACTGACC TAAGCTGGAACAGACG
34	TCCGACACGTCCGATT GATGGCATTGGTGGATTAAGCGG atgg tgagc aaggg cgagg aggat aacat ggcca CCTGACCGGCT CCACCACTGACC TAAGCTGGAACAGACG
35	TCCGACACGTCCGATT GATGGCATTGGTGGATTAAGCGG atgg tgagc aaggg cgagg aggat aacat ggcca t CCTGACCGGCT CCACCACTGACC TAAGCTGGAACAGACG
36	TCCGACACGTCCGATT GATGGCATTGGTGGATTAAGCGG atgg tgagc aaggg cgagg aggat aacat ggcca tc CCTGACCGGCT CCACCACTGACC TAAGCTGGAACAGACG
37	TCCGACACGTCCGATT GATGGCATTGGTGGATTAAGCGG atgg tgagc aaggg cgagg aggat aacat ggcca tca CCTGACCGGCT CCACCACTGACC TAAGCTGGAACAGACG
38	TCCGACACGTCCGATT GATGGCATTGGTGGATTAAGCGG atgg tgagc aaggg cgagg aggat aacat ggcca tcat CCTGACCGGCT CCACCACTGACC TAAGCTGGAACAGACG
39	TCCGACACGTCCGATT GATGGCATTGGTGGATTAAGCGG atgg tgagc aaggg cgagg aggat aacat ggcca tcatc CCTGACCGGCT CCACCACTGACC TAAGCTGGAACAGACG
44	TCCGACACGTCCGATT GATGGCATTGGTGGATTAAGCGG atgg tgagc aaggg cgagg aggat aacat ggcca tcatc aagga CCTGACCGGCT CCACCACTGACC TAAGCTGGAACAGACG

49	TCCGACACGTCCGATT GATGGCATTGGTGGATTAAGCGG atgg tgagc aaggg cgagg aggat aacat ggcca tcatc aagga gttca CCTGACCGGCT CCACCACTGACC TAAGCTGGAACAGACG
54	TCCGACACGTCCGATT GATGGCATTGGTGGATTAAGCGG atgg tgagc aaggg cgagg aggat aacat ggcca tcatc aagga gttca tgcgc CCTGACCGGCT CCACCACTGACC TAAGCTGGAACAGACG
59	TCCGACACGTCCGATT GATGGCATTGGTGGATTAAGCGG atgg tgagc aaggg cgagg aggat aacat ggcca tcatc aagga gttca tgcgc ttcaa CCTGACCGGCT CCACCACTGACC TAAGCTGGAACAGACG
64	TCCGACACGTCCGATT GATGGCATTGGTGGATTAAGCGG atgg tgagc aaggg cgagg aggat aacat ggcca tcatc aagga gttca tgcgc ttcaa ggtgc CCTGACCGGCT CCACCACTGACC TAAGCTGGAACAGACG
69	TCCGACACGTCCGATT GATGGCATTGGTGGATTAAGCGG atgg tgagc aaggg cgagg aggat aacat ggcca tcatc aagga gttca tgcgc ttcaa ggtgc acatg CCTGACCGGCT CCACCACTGACC TAAGCTGGAACAGACG
74	TCCGACACGTCCGATT GATGGCATTGGTGGATTAAGCGG atgg tgagc aaggg cgagg aggat aacat ggcca tcatc aagga gttca tgcgc ttcaa ggtgc acatg gaggg CCTGACCGGCT CCACCACTGACC TAAGCTGGAACAGACG
79	TCCGACACGTCCGATT GATGGCATTGGTGGATTAAGCGG atgg tgagc aaggg cgagg aggat aacat ggcca tcatc aagga gttca tgcgc ttcaa ggtgc acatg gaggg ctccg CCTGACCGGCT CCACCACTGACC TAAGCTGGAACAGACG
84	TCCGACACGTCCGATT GATGGCATTGGTGGATTAAGCGG atgg tgagc aaggg cgagg aggat aacat ggcca tcatc aagga gttca tgcgc ttcaa ggtgc acatg gaggg ctccg tgaac CCTGACCGGCT CCACCACTGACC TAAGCTGGAACAGACG
89	TCCGACACGTCCGATT GATGGCATTGGTGGATTAAGCGG atgg tgagc aaggg cgagg aggat aacat ggcca tcatc aagga gttca tgcgc ttcaa ggtgc acatg gaggg ctccg tgaac ggcca CCTGACCGGCT CCACCACTGACC TAAGCTGGAACAGACG
154	TCCGACACGTCCGATT GATGGCATTGGTGGATTAAGCGG atgg tgagc aaggg cgagg aggat aacat ggcca tcatc aagga gttca tgcgc ttcaaggtgcacatggagggctccgtgaacggccacgagttcgagatcgagggcgagggcgagggccgcccctac gagggcacccagaccgccaagctga CCTGACCGGCT CCACCACTGACC TAAGCTGGAACAGACG
711	TCCGACACGTCCGATT GATGGCATTGGTGGATTAAGCGG atggtgagcaagggcgaggaggataacatggccatcatcaaggagttcatgcgcttcaaggtgcacatggagggctccgtgaacggccacgagttcgagatcgagggcgagggcgagggccgcccctacgagggcacccagaccgccaagctgaaggtgaccaagggtggccccctgcccttcgcctgggacatcctgtcccctcagttcatgtacggctccaaggcctacgtgaagcaccccgccgacatccccgactacttgaagctgtccttccccgagggcttcaagtgggagcgcgtgatgaacttcgaggacggcggcgtggtgaccgtgacccaggactcctccctgcaggacggcgagttcatctacaaggtgaagctgcgcggcaccaacttcccctccgacggccccgtaatgcagaagaagaccatgggctgggaggcctcctccgagcggatgtaccccgaggacggcgccctgaagggcgagatcaagcagaggctgaagctgaaggacggcggccactacgacgctgaggtcaagaccacctacaaggccaagaagcccgtgcagctgcccggcgcctacaacgtcaacatcaagttggacatcacctcccacaacgaggactacaccatcgtggaacagtacgaacgcgccgagggccgccactccaccggcggcatggacgagctgtacaagta CCTGACCGGCT CCACCACTGACC TAAGCTGGAACAGACG
