# Supplementary material for: A dCas9 proximity reporter for investigating DNA linear and rotational dynamics
Source: PLoS One. 2026 Oct 29;21(10):e0355748. doi: 10.1371/journal.pone.0355748 (PMC13719016; doi:10.1371/journal.pone.0355748)
Supplement: Supplementary file 5 [file pone.0355748.s005.pdf]

**S5 Table. DNA Sequences for the PAM-OUT Orientation**	
**Base Separation**	**DNA Sequence**
40	TCCGACACGTCCGATTCCGCTTAATCCACCAATGCCATCGGTCAGTGGTGGAGCCGGTCAGGTAAGCTGGAACAGACG
41	TCCGACACGTCCGATTCCGCTTAATCACCAATGCCATCAGGTCAGTGGTGGAGCCGGTCAGGTAAGCTGGAACAGACG
42	TCCGACACGTCCGATTCCGCTTAATCCACCAATGCCATCACGGTCAGTGGTGGAGCCGGTCAGGTAAGCTGGAACAGACG
43	TCCGACACGTCCGATTCCGCTTAATCCACCAATGCCATCACTGGTCAGTGGTGGAGCCGGTCAGGTAAGCTGGAACAGACG
44	TCCGACACGTCCGATTCCGCTTAATCCACCAATGCCATCACTGGGTCAGTGGTGGAGCCGGTCAGGTAAGCTGGAACAGACG
45	TCCGACACGTCCGATTCCGCTTAATCCACCAATGCCATCACTGAGGTCAGTGGTGGAGCCGGTCAGGTAAGCTGGAACAGACG
46	TCCGACACGTCCGATTCCGCTTAATCCACCAATGCCATCACTGACGGTCAGTGGTGGAGCCGGTCAGGTAAGCTGGAACAGACG
47	TCCGACACGTCCGATTCCGCTTAATCCACCAATGCCATCACTGACTGGTCAGTGGTGGAGCCGGTCAGGTAAGCTGGAACAGACG
48	TCCGACACGTCCGATTCCGCTTAATCCACCAATGCCATCACTGACTCGGTCAGTGGTGGAGCCGGTCAGGTAAGCTGGAACAGACG
49	TCCGACACGTCCGATTCCGCTTAATCCACCAATGCCATCACTGACTCAGGTCAGTGGTGGAGCCGGTCAGGTAAGCTGGAACAGACG
50	TCCGACACGTCCGATTCCGCTTAATCCACCAATGCCATCACTGACTCACGGTCAGTGGTGGAGCCGGTCAGGTAAGCTGGAACAGACG
