# Supplementary material for: A dCas9 proximity reporter for investigating DNA linear and rotational dynamics
Source: PLoS One. 2026 Oct 29;21(10):e0355748. doi: 10.1371/journal.pone.0355748 (PMC13719016; doi:10.1371/journal.pone.0355748)
Supplement: Supplementary file 6 [file pone.0355748.s006.pdf]

S6 Table. DNA Sequence for the gRNA Templates	
**gRNA Construct**	**DNA Sequence**
SP6-spacer-2xMS2	atttaggtgacactatagatggcattggtggattaaggtttaagagctatgctggaaacagcatagcaagtttaaataaggctagtccgttatcaacttgaaaaagtggcaccgagtcggtgcgggagcacatgaggatcacccatgtgccacgagcgacatgaggatcacccatgtcgctcgtgttcccttttttt
SP6-spacer-2xPP7	atttaggtgacactataggtcagtggtggagccggtcgtttaagagctatgctggaaacagcatagcaagtttaaataaggctagtccgttatcaacttgaaaaagtggcaccgagtcggtgcgggagctaaggagtttatatggaaacccttagcctgctgcgtaaggagtttatatggaaacccttacgcagcagttcccttttttt
SP6-spacer-8xMS2-end	atttaggtgacactatagatggcattggtggattaaggtttaagagctatgctggaaacagcatagcaagtttaaataaggctagtccgttatcaacttgaaaaagtggcaccgagtcggtgcctgccatgaggaatgaccaccaggcattccgatccgacgatggaccatcaggccatcgagctgcagaagtgacgaccacgcacttcggagtgacagaggaggatcacccctctggccaccagagtagagcatcagcctactccggacaactacggaggaccaccccgtaggagtagagcgaggagcaccagccctcgcgtgtgacgatgacgatcacgcatcgtcacacaaactactcaaatgtccgaaaggtggcaaacactccaaagcagctaaacggatgaaacatggcactttttttt
SP6-spacer-8xPP7-end	atttaggtgacactataggtcagtggtggagccggtcgtttaagagctatgctggaaacagcatagcaagtttaaataaggctagtccgttatcaacttgaaaaagtggcaccgagtcggtgcgggactgccatgaggagcagacgatatggcgtcgctccgatccgataaggagtttatatggaaacccttaagctgcaggaccagaagatatggcttcggtccggagtgaggaccagaagatatgacttcggtccgccaccacctccactgtgtagggacagggaggcggacaacctggagaatgtagggattcccagggagtagaattccacaatgtagggattgggaatgtgtgaagctggagaatgtagggattcccagctcacacaaactactcaaatgtccgaaaggtggcaaacactccaaagcagctaaacggatgaaacatggcacccctttttttt
SP6-spacer-8xMS2-tetraloop	atttaggtgacactatagatggcattggtggattaaggtttgagagctaggccctgccatgaggaatgaccaccaggcattccgatccgacgatggaccatcaggccatcgagctgcagaagtgacgaccacgcacttcggagtgacagaggaggatcacccctctggccaccagagtagagcatcagcctactccggacaactacggaggaccaccccgtaggagtagagcgaggagcaccagccctcgcgtgtgacgatgacgatcacgcatcgtcacacaaactactcaaatgtccgaaaggtggcaaacactccaaagcagctaaacggatgaaacatggcacggcctagcaagttcaaataaggctagtccgttatcaacttgaaaaagtggcaccgagtcggtgcttttttt
SP6-spacer-8xPP7-tetraloop	atttaggtgacactataggtcagtggtggagccggtcgtttgagagctaccctgccatgaggagcagacgatatggcgtcgctccgatccgataaggagtttatatggaaacccttaagctgcaggaccagaagatatggcttcggtccggagtgaggaccagaagatatgacttcggtccgccaccacctccactgtgtagggacagggaggcggacaacctggagaatgtagggattcccagggagtagaattccacaatgtagggattgggaatgtgtgaagctggagaatgtagggattcccagctcacacaaactactcaaatgtccgaaaggtggcaaacactccaaagcagctaaacggatgaaacatggcacggtagcaagttcaaataaggctagtccgttatcaacttgaaaaagtggcaccgagtcggtgcttttttt
