# Supplementary material for: A dCas9 proximity reporter for investigating DNA linear and rotational dynamics
Source: PLoS One. 2026 Oct 29;21(10):e0355748. doi: 10.1371/journal.pone.0355748 (PMC13719016; doi:10.1371/journal.pone.0355748)
Supplement: Supplementary file 7 [file pone.0355748.s007.pdf]

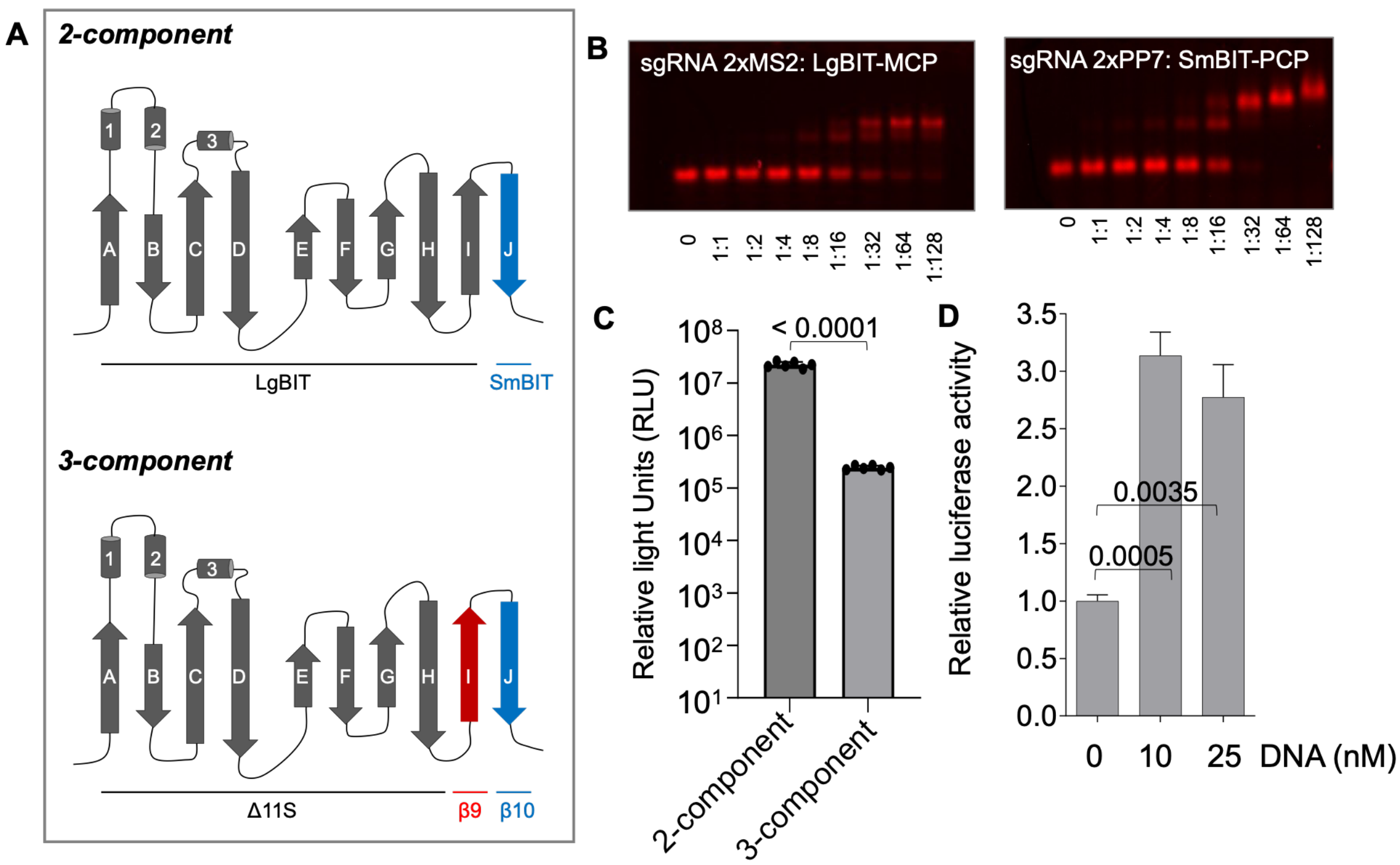

S1 Fig. A. Schematics of the 2-component vs the 3-component split NanoLuc ® system. **B.** The fusion protein consisting of LgBIT-MCP and SmBIT-PCP bind to gRNAs fused to 2xMS2 and 2xPP7 aptamers, respectively. **C.** The 2-component system has a higher background signal than the 3-component system likely due to spontaneous complementation of the luciferase components. The assay components included: 2-component system (50 nM LgBIT-MCP, 50 nM SmBIT-PCP, 25 nM each of dCas9 and gRNA aptamers, and 10 µM substrate); 3-component (50 nM Δ11S, 500 nM each of β9-PCP and β10-MCP , 25 nM each of dCas9 and gRNA aptamers, and 10 µM substrate. **D.** In vitro complementation assay using PAM-IN DNA where the two target sites are separated by 34 bp results in a 2.5-3-fold increase in luciferase signal. Increasing the DNA concentration does not significantly change the fold change in luciferase activity. N =3. Data is presented as Mean +/- SEM.
