# Supplementary material for: A dCas9 proximity reporter for investigating DNA linear and rotational dynamics
Source: PLoS One. 2026 Oct 29;21(10):e0355748. doi: 10.1371/journal.pone.0355748 (PMC13719016; doi:10.1371/journal.pone.0355748)
Supplement: Supplementary file 8 [file pone.0355748.s008.pdf]

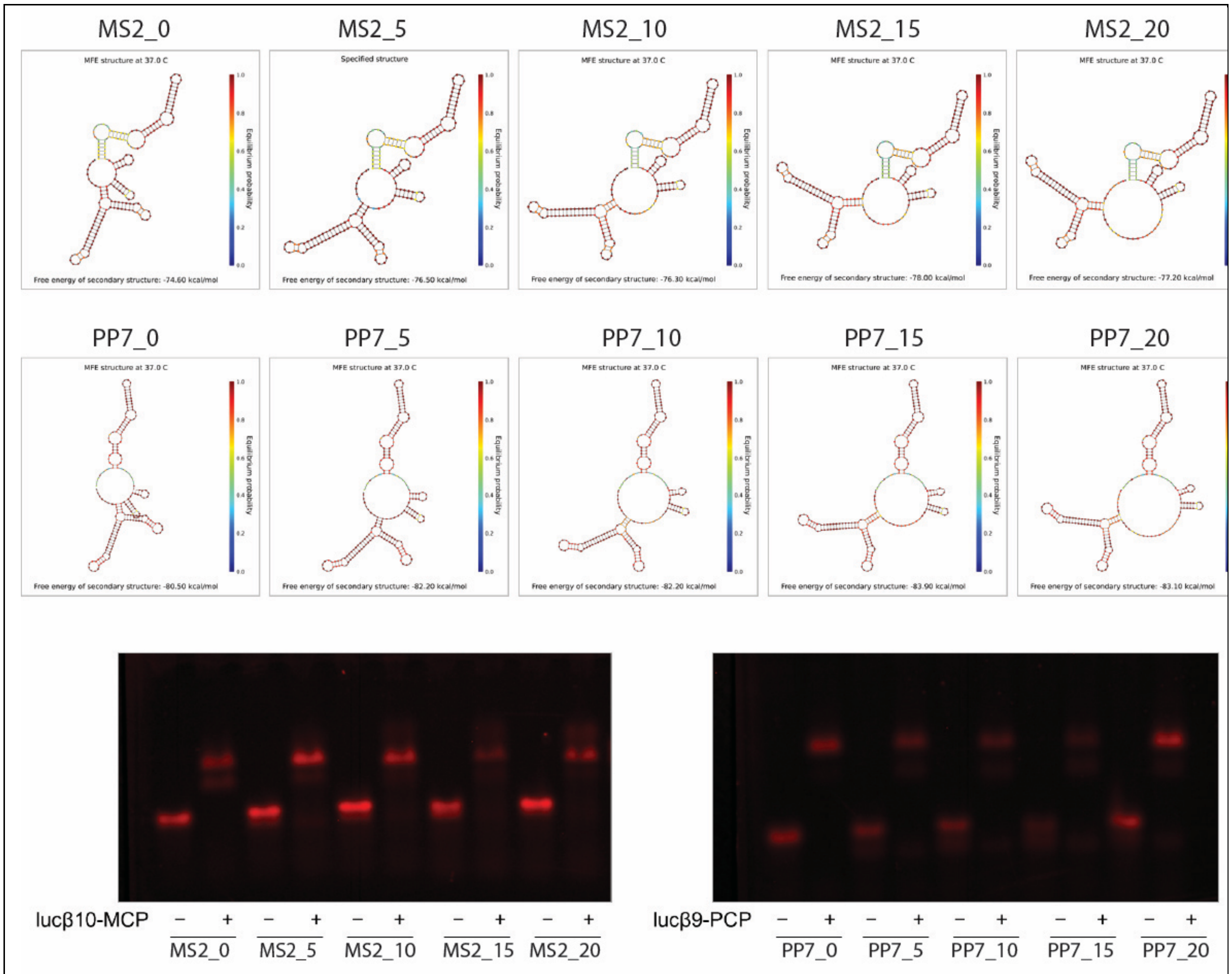

S2 Fig. RNA aptamer modified gRNA structures and their binding with fusion proteins A. NUPACK-predicted structures of gRNA modified with 2x tandem MS2 and PP7 RNA aptamer with varying linker lengths. Numbers indicate the linker length (number of nucleotides) in between the gRNA backbone and the RNA aptamer. **B.** EMSA of the fusion proteins binding to the different gRNA-aptamer constructs.
