# Supplementary material for: A dCas9 proximity reporter for investigating DNA linear and rotational dynamics
Source: PLoS One. 2026 Oct 29;21(10):e0355748. doi: 10.1371/journal.pone.0355748 (PMC13719016; doi:10.1371/journal.pone.0355748)
Supplement: Supplementary file 9 [file pone.0355748.s009.pdf]

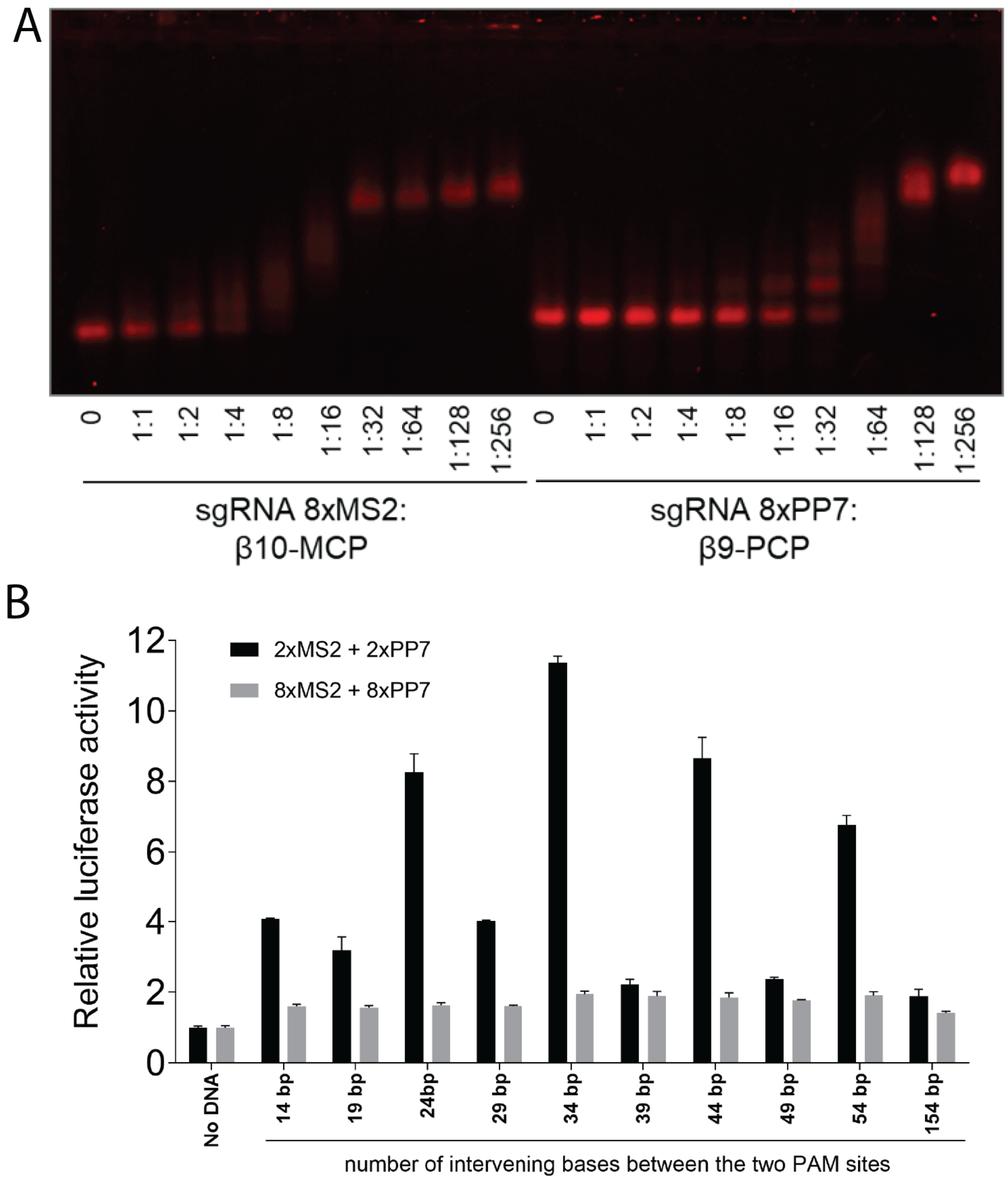

S3 Fig. In vitro complementation assay with gRNAs modified with eight tandem RNA aptamers in the tetraloop A. An EMSA of fusion proteins binding with gRNAs modified with eight tandem RNA aptamers within the tetraloop. **B.** Reporter activity with gRNAs modified with eight tandem repeats of the RNA aptamer in the tetraloop showed no signal whereas gRNAs with two tandem repeats of the aptamers appended at the end of the gRNA showed 10-12 fold reporter activity. Data are presented as Mean ± SEM (N = 3) for all samples.
