# Supplementary material for: A dCas9 proximity reporter for investigating DNA linear and rotational dynamics
Source: PLoS One. 2026 Oct 29;21(10):e0355748. doi: 10.1371/journal.pone.0355748 (PMC13719016; doi:10.1371/journal.pone.0355748)
Supplement: Supplementary file 10 [file pone.0355748.s010.pdf]

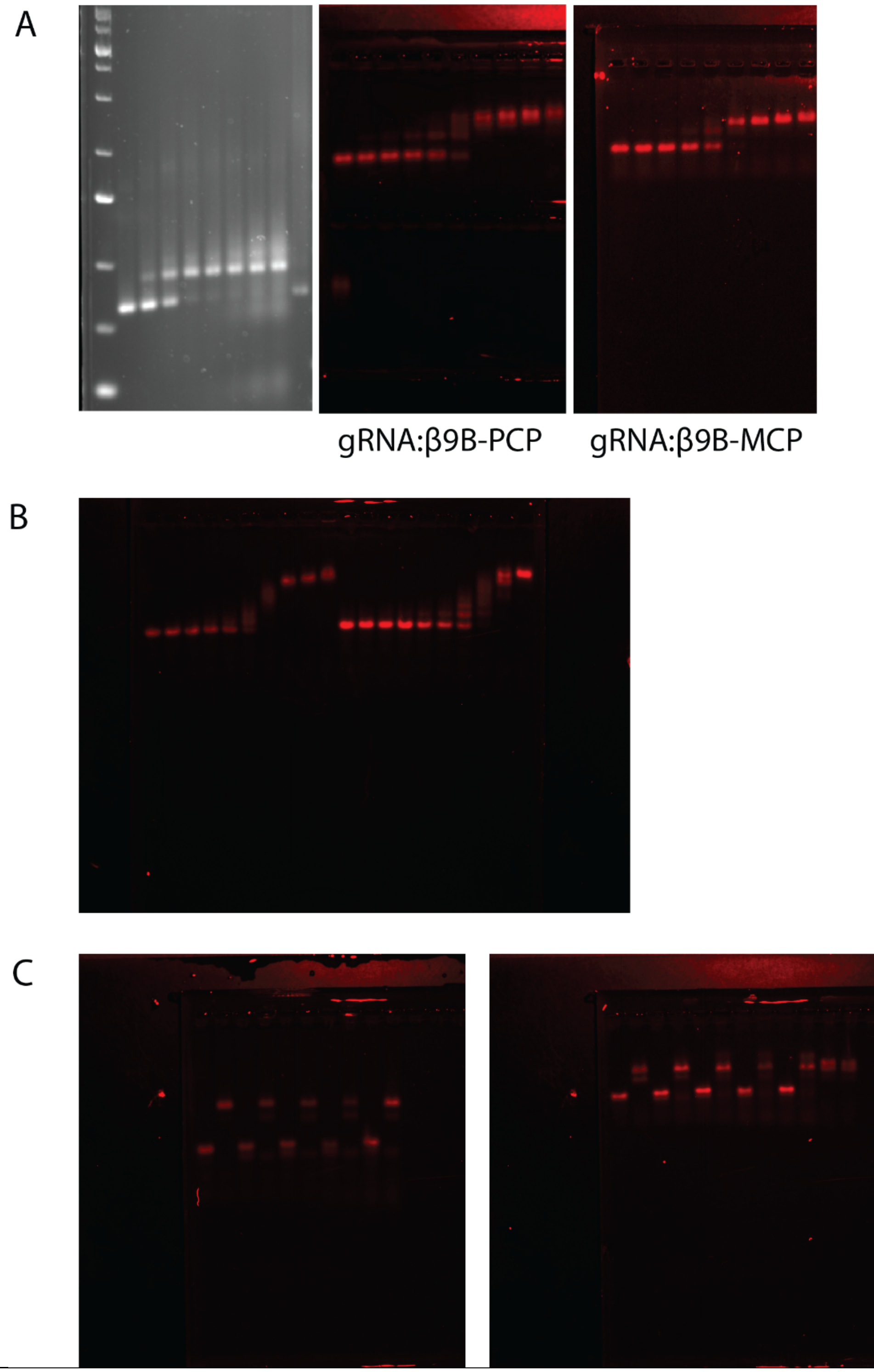

S4 Fig. Full gel images A. Full gels from Fig 2B and 2C. **B.** Full gels from Fig 4. **C** Full gels from Fig S1
